# Use of digital teaching resources and predictors of medical student performance during the pandemic: A prospective study

**DOI:** 10.1371/journal.pone.0268331

**Published:** 2022-05-11

**Authors:** Michelle Seer, Charlotte Kampsen, Tim Becker, Sebastian Hobert, Sven Anders, Tobias Raupach

**Affiliations:** 1 Institute of Medical Education, University Hospital Bonn, Bonn, Germany; 2 Department of Cardiology and Pneumology, Göttingen University Medical Centre, Göttingen, Germany; 3 Division of Medical Education, Göttingen University Medical Centre, Göttingen, Germany; 4 Division of Application Systems and E-Business, University of Göttingen, Göttingen, Germany; 5 Campus Institute Data Science, Göttingen, Germany; 6 Department of Legal Medicine, University Medical Centre Hamburg-Eppendorf, Hamburg, Germany; Osaka Medical Center for Cancer and Cardiovascular Diseases, JAPAN

## Abstract

**Background:**

The coronavirus pandemic has led to increased use of digital teaching formats in medical education. A number of studies have assessed student satisfaction with these resources. However, there is a lack of studies investigating changes in student performance following the switch from contact to virtual teaching. Specifically, there are no studies linking student use of digital resources to learning outcome and examining predictors of failure.

**Methods:**

Student performance before (winter term 2019/20: contact teaching) and during (summer term 2020: no contact teaching) the pandemic was compared prospectively in a cohort of 162 medical students enrolled in the clinical phase of a five-year undergraduate curriculum. Use of and performance in various digital resources (case-based teaching in a modified flipped classroom approach; formative key feature examinations of clinical reasoning; daily multiple choice quizzes) was recorded in summer 2020. Student scores in summative examinations were compared to examination scores in the previous term. Associations between student characteristics, resource use and summative examination results were used to identify predictors of performance.

**Results:**

Not all students made complete use of the digital learning resources provided. Timely completion of tasks was associated with superior performance compared to delayed completion. Female students scored significantly fewer points in formative key feature examinations and digital quizzes. Overall, higher rankings within the student cohort (according to summative exams) in winter term 2019/20 as well as male gender predicted summative exam performance in summer 2020. Scores achieved in the first formative key feature examination predicted summative end-of-module exam scores.

**Conclusions:**

The association between timely completion of tasks as well as early performance in a module and summative exams might help to identify students at risk and offering help early on. The unexpected gender difference requires further study to determine whether the shift to a digital-only curriculum disadvantages female students.

## Introduction

The Covid-19 pandemic has impacted medical education globally: In many countries, all teaching activities had to be replaced with digital formats [1]. While it is hard to find a surrogate for patient encounters in medical education, alternatives to traditional classroom teaching focusing on the acquisition and application of knowledge have been available for some time. For instance, the effectiveness of the flipped classroom approach has been studied extensively [2]; other digital interventions (e.g., VR-based anatomy teaching [3], and machine learning applications [4]) have been developed. Some of these were introduced to medical curricula prior to formal research showing their effectiveness regarding student learning outcome, and owing to the high urgency situation not all of these formats were grounded in educational psychology. Published research indicates that a complete conversion to a digital-only course may not entail smaller student learning outcome in first-year medical students [5]. One study comparing summative exam data in the clinical phase of an undergraduate medical curriculum between winter term 2019/20 (before the pandemic) and summer term 2020 (switch to digital learning due to the pandemic) did not find a statistically significant difference between the performance of the respective student cohorts [6]. The majority of studies on medical education during the corona pandemic is descriptive in nature [7]. At the same time, it would be important to know in which way student engagement with digital teaching resources impacts on knowledge acquisition and performance in summative examinations. More specifically, factors associated with (un)successful adaption to virtual teaching need to be identified in order to be able to support students at risk [8].

The aims of this study were (1) to compare student rankings within their cohort during contact restrictions to rankings within the same cohort prior to the pandemic, (2) to investigate the association between the usage of available learning resources and learning outcome at the end of term, and (3) to identify early predictors of less favourable end-of-term performance (students at risk). With regard to the second study aim, we hypothesised that intensive use of resources and early completion of digital tasks as well as better performance in these tasks is associated with more favourable outcome in a subsequent summative examination.

## Methods

### Educational setting

This prospective cohort study was conducted at Göttingen University Medical Centre in summer term 2020. Undergraduate medical education consists of a two-year pre-clinical and a three-year clinical phase. The latter adopts a modular structure, and at the beginning of year 4, all students take a six-week module on cardiovascular and respiratory disease. The overarching learning objectives of this module comprise aetiology, pathophysiology, signs and symptoms, investigations and treatment options relevant to these specialties. Given that the module focuses on the acquisition of knowledge and clinical reasoning, the main teaching formats include lectures, case-based small group sessions, and bedside teaching. Before the pandemic, a number of digital resources were available: In weekly electronic case seminars (e-seminars), students individually worked on patient cases that were augmented with key feature questions. Utilising the direct testing effect [9], these seminars were designed to foster clinical reasoning competence [10, 11]. In order to support the acquisition of factual knowledge and to foster continuous repetition of material, students were provided with an app (‘#clue’) presenting two high-stakes exam questions per day; questions were aligned to the lecture content of the preceding day and were repeated in order to facilitate spaced testing [12]. More recently, a virtual accident and emergency department was introduced to the module. In this serious game that is routed in self-determination theory [13], students take on the role of attending physicians and need to diagnose and treat virtual patients. This approach has been shown to have a sustained effect on clinical reasoning competence [14]. These three digital teaching interventions were also used during the pandemic. Lectures and case-based small-group teaching were transformed into modified flipped classroom sessions (see below).

### Changes to the structure of the module

**Fig 1:**
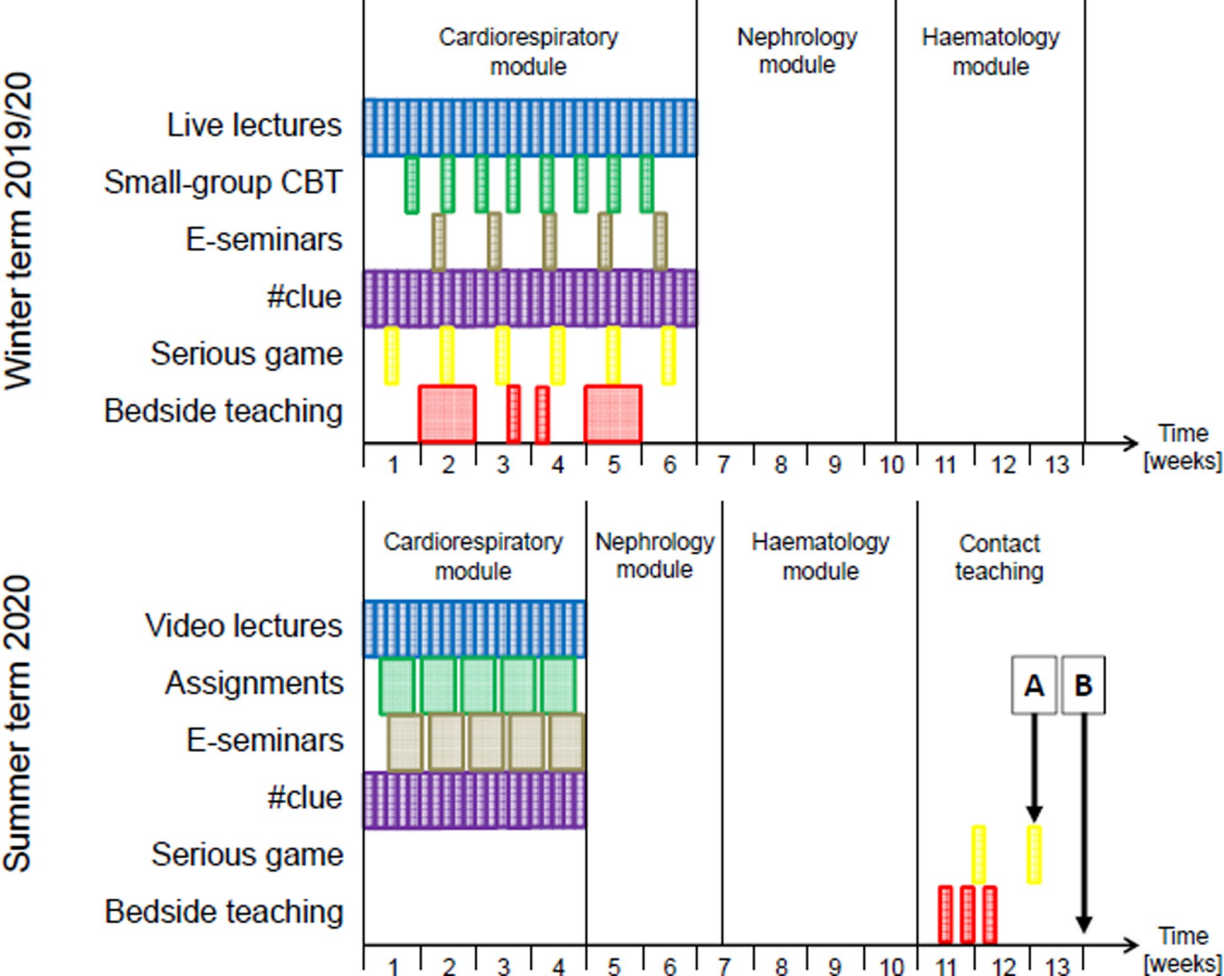
Timeline of teaching before (winter term 2019/20) and after the start of the pandemic (summer term 2020). CBT, case-based teaching. A, data collection point for the formative serious game assessment; B, summative end-of-term examination.

In summer term 2020, teaching formats aiming at knowledge acquisition and application were separated from practical training because educators at our institution hoped that contact teaching would be possible in June/July. As a consequence, the six-week cardiorespiratory module in year 4 was shortened to four weeks during which students participated in modified flipped classroom sessions as well as in electronic case seminars (distance learning). They were also invited to use the #clue app (see below). As a consequence of shortening the cardiorespiratory and the two subsequent modules (nephrology/rheumatology and haematology/oncology), a three-week period for contact teaching was created at the end of term during which each student received a total of 15 hours of bedside teaching in adult and paediatric cardiology as well as thoracic surgery and pneumology. In addition, students received one 90-minute introductory session of the serious game and took part in a second 90-minute session during which up to 8 virtual patient cases were displayed (Fig 1). Despite these sessions being held in a classroom equipped with computers, the instructional format was still digital in nature, and each student worked on their own cases (see below). In this regard, the serious game did not represent proper contact teaching.

The content, mode of delivery and user data generated by the components of the new digital module are described in more detail below. Educational targets (factual knowledge or knowledge application / clinical reasoning) are given for each digital learning resource.

#### Modified flipped classroom sessions and assignments

Lectures were recorded using Camtasia Studio 8 (TechSmith) and made available to students via the institution’s content management system Stud.IP. Compared to the traditional module, the number of lectures remained unchanged. Neither lecture attendance (before the pandemic) nor resource use (summer term 2020) was mandatory. For this reason, student behaviour on the platform was not tracked, and no data on viewings of the material were available. In the traditional module, lecture content would serve as the basis for eight 90-minute sessions of small-group, case-based learning. In these sessions, students used to work through a total of five paper cases (myocardial infarction, heart failure with atrial fibrillation, atrial septal defect, chronic obstructive pulmonary disease, and pulmonary embolism) under the supervision of a clinical teacher. In the ‘modified flipped classroom’ paradigm, students were asked to watch lecture recordings and work on the same five cases that were provided as email attachments. However, instead discussing these in small groups, they were provided with a number of essay questions (‘assignments’) on patient management (educational target: knowledge application / clinical reasoning). Students worked on the cases on their own; completed assignments were to be sent to a tutor via email, and each student received individual feedback on their performance. Essays were not graded but information on the delay between the provision of each assignment and submission of the essay was available for every student.

#### Electronic case seminars

As stated above, these seminars were designed to foster clinical reasoning. Each session was made up of four patient cases that were displayed on the institution’s learning management platform. Each of these contained five key feature questions on diagnosis and treatment to be answered using a long menu (for more detail see [11]). Content was aligned to the order in which lectures were made available on the learning management system. In contrast to the traditional module, students did not gather in the institution’s computer resource facilities in order to complete e-seminars. Instead, these seminars were made available through an online learning platform (ILIAS). Five e-seminars containing a total of 20 cases with a maximum score of 100 points were provided, and students were strongly recommended to complete these on a voluntary basis. In order to avoid cramming, e-seminars were only available for a limited period of time (three to eight days). Comprehensive data including the date and time of case completion, the delay between provision of each e-seminar on ILIAS and the date of completion as well as the points scored in each e-seminar were collected for individual students.

#### #clue

This app aiming at ‘cross-linking undergraduate education’ (clue) presented students with two multiple choice (MC) questions per day. Questions were taken from previous high-stakes examinations and aligned to the content discussed in preceding lectures. The concept underlying #clue was that by activating knowledge shortly after its acquisition, long-term retention might be enhanced (educational target: knowledge acquisition and retention). In addition, students might feel less pressure before high-stakes examinations if they have already worked with similar questions through the clinical phase of undergraduate education. During the four-week digital module, a total of 40 questions were presented. Each question was available for ten days; items that were answered within that period were stored and remained visible for students. If left unanswered, they disappeared from the list. According to an earlier study using this approach [15], this was expected to increase motivation to use the app as intended (i.e., continuous repetition) as opposed to using it for cramming before the final exam. Automated feedback on the correct answer option was given directly after each question. There was also a dashboard allowing students to check their own performance against the entire cohort. For each student, extensive data on app use were available, including how many questions were answered, how many of these were answered correctly, the date (and delay following release of a question in the app) as well as the time of day each question was answered.

### Measurement of learning outcome

Lecture recordings and #clue were intended to support knowledge acquisition and retention whereas e-seminars and assignments within the modified flipped classroom approach aimed at fostering knowledge application / clinical reasoning. Thus, in order to assess learning outcome, two different examination formats were used.

**Factual knowledge** was tested by means of a summative MC exam at the end of term (i.e., following the other two modules and the three-week period of contact teaching). It consisted of 45 questions about adult and paediatric cardiology, thoracic surgery, and pneumology. Questions were written by experienced faculty; a panel of teachers assessed whether items were adequately structured and face valid. Students sat a paper-and-pencil exam, results were retrieved from the central assessment office, and raw scores were converted to percentages. In order to compare student performance in this exam to previous general performance levels (unrelated to cardiology and pneumology), the percent score of points achieved in all summative exams in the preceding term was obtained for all students and converted to percentile ranks.

**Knowledge application** was assessed using the virtual accident and emergency department. While this serious game was originally designed for teaching purposes, student performance regarding patient management can also be judged by analysing the logfiles of in-game activity (for detail see [16]). The second 90-minute gaming session served at the data collection point. Students were presented with a number of virtual patients in random order. However, the session was designed in such a way that each student saw two pre-defined virtual patients suffering from community-acquired pneumonia and infective endocarditis. The game prompted students to take a history, order laboratory and other diagnostic tests, make a diagnosis, initiate treatment, and transfer patients to the most appropriate care unit within the virtual hospital. A checklist specifically designed for this purpose was used to assess student performance; each appropriate action (as saved in the game logfile for each student) generated one raw point for a total of 31 points for the two virtual patients; raw scores were converted to percentages. Performance in the gaming session was not formally graded (formative examination).

### Ethics approval and consent to participate

This study was approved by the local Ethics Committee (Ethik-Kommission der Universitätsmedizin Göttingen; application number 38/7/20). Students were informed about the rationale and aims of the study by e-mail, Study participation was voluntary and all participants gave their written consent by signing an informed consent form before entering the study. We made every effort to comply with data protection rules and all data were anonymized prior to analysis. Methods were carried out in accordance with relevant guidelines and regulations.

### Statistical analysis

Data obtained from the various learning resources as well as examination results were merged by student identifier codes and imported into IBM SPSS Statistics 25.0 (IBM Corp, Armonk, NY, USA). First, descriptive analyses of resource use and student performance were conducted, taking student gender and previous performance levels into account. Subsequent exploratory analyses addressed the three aims stated above:

Analysis 1) In order to compare student performance before and after the switch to an entirely digital curriculum, percentile ranks within the student cohort based on scores achieved in summative MC examinations in the current and the preceding term were calculated, plotted and–according to findings of earlier studies [17]–categorised by student gender.

Analysis 2) In order to investigate the association between the usage of available learning resources and learning outcome at the end of term, univariate and multivariate linear regression models were run with performance in the end-of-term exam (for knowledge retention) and performance in the serious game (for knowledge application) as dependent variables, respectively. We hypothesised that more intensive/successful use of digital teaching formats designed to foster knowledge acquisition and retention would be associated with better results in the multiple choice exam while more intensive/successful use of resources intended to enhance knowledge application would be associated with superior performance in the serious game.

Analysis 3) In order to identify early predictors of unfavourable exam performance, univariate and multivariate linear regression models were run with percent score in the MC exam as the dependent variable and student behaviour with regard to the first assignment, the first e-seminar and the first 10 #clue items as independent variables.

Data are reported as mean ± standard deviation, proportions, ranks and effect sizes (Cohen’s d [18]). T Tests were conducted to assess between-group differences. For linear regressions, beta coefficients (95% confidence interval, CI) are given as well as variance explained (R^2^). Significance levels were set to 5%.

## Results

### Student characteristics

A total of 162 out of 169 students enrolled in the module provided written consent to participate in the study (response rate 95.9%; sample size varies due to missing data for individual analyses). The proportion of female students was 67.9%. Students were aged 24.8 ± 3.5 years.

### Use of learning resources

#### Assignments

There were five assignments, and mean delay between provision of tasks on the learning management system and submission of essays decreased over time (5.6 ± 5.8 days; 5.8 ± 4.1 days; 4.2 ± 3.0 days; 3.5 ± 2.3 days; 2.9 ± 3.4 days). Just over half of all students (53.4%) completed at least three assignments earlier than 50% of the cohort; these students were labelled as ‘early completers’ for subsequent analyses. There was no significant difference between early and non-early completers with regard to percentile ranks according to exams in the previous term (early completers: 54.3 ± 27.8; non-early completers: 50.4 ± 28.7; p = 0.390).

#### E-seminars

About half of all students (51.9%) completed all five e-seminars, and another 29% completed three or four. Only 16 students (9.9%) did not use this resource at all. Students who completed at least one e-seminar achieved a mean of 67.5 ± 11.0% of all available points. With the exception of the first e-seminar (mean delay between release and completion: 3.1 ± 2.3 days), a majority of students completed e-seminars on the day they were made available on the learning management system or on the following day. Just under half of all students (45.9%) completed at least three e-seminars earlier than 50% of their cohort; these students were labelled as ‘early completers’, and they achieved a significantly higher percent score compared to non-early completers (70.8 ± 10.2% vs. 64.7 ± 10.9%; p = 0.001; d = 0.57). Regardless of early or non-early completion, female students scored significantly fewer points than male students (65.6 ± 10.8% vs. 72.2 ± 10.1%; p = 0.001; d = 0.62). Overall, there was also a weak but positive correlation between percentile rank in previous exams and the percent score achieved in e-seminars (r = 0.199; p = 0.018).

#### #clue

A majority of 129 students (79.6%) made at least one attempt to answer any of the 40 questions provided; in this group, the mean number of questions answered was 29.0 ± 13.3, with 41.9% of students answering all 40 questions. Overall, 70.5 ± 13.4% of questions were answered correctly. Mean delay between the release of questions and completion by students was 2.2 ± 1.9 days. Students who completed questions on the day they were put online or on the following day were labelled as ‘early completers’ (61.2%), and they provided significantly more correct answers than their peers (73.6 ± 14.6% vs. 65.6 ± 9.6%; p = 0.001; d = 0.63). Female students answered more questions than male students (30.7 ± 11.9 vs. 25.5 ± 15.5; p = 0.037; d = 0.40); however, they scored significantly lower than males (68.3 ± 12.9 vs. 75.3 ± 13.5%; p = 0.005; d = 0.54). Within the entire cohort, percentile rank in previous exams and percentage of #clue questions answered correctly were positively correlated (r = 0.278; p = 0.002).

### Learning outcome

Internal consistency of the summative MC examination was acceptable (Cronbach’s α = 0.698). Students scored 86.4 ± 8.4% of available points, and male students achieved significantly higher scores than female students (89.2 ± 7.3 vs. 85.1 ± 8.6; p = 0.004; d = 0.46). Internal consistency of the formative serious gaming session was acceptable (Cronbach’s α = 0.717). Students achieved a mean score of 47.6 ± 14.0% with no significant difference between male and female students (46.5 ± 16.0% vs. 48.2 ± 13.0%; p = 0.503). There was no significant correlation between percent scores in the summative MC exam and the formative gaming session, indicating that the two formats measured different constructs.

### Analysis 1: Performance trajectories

**Fig 2:**
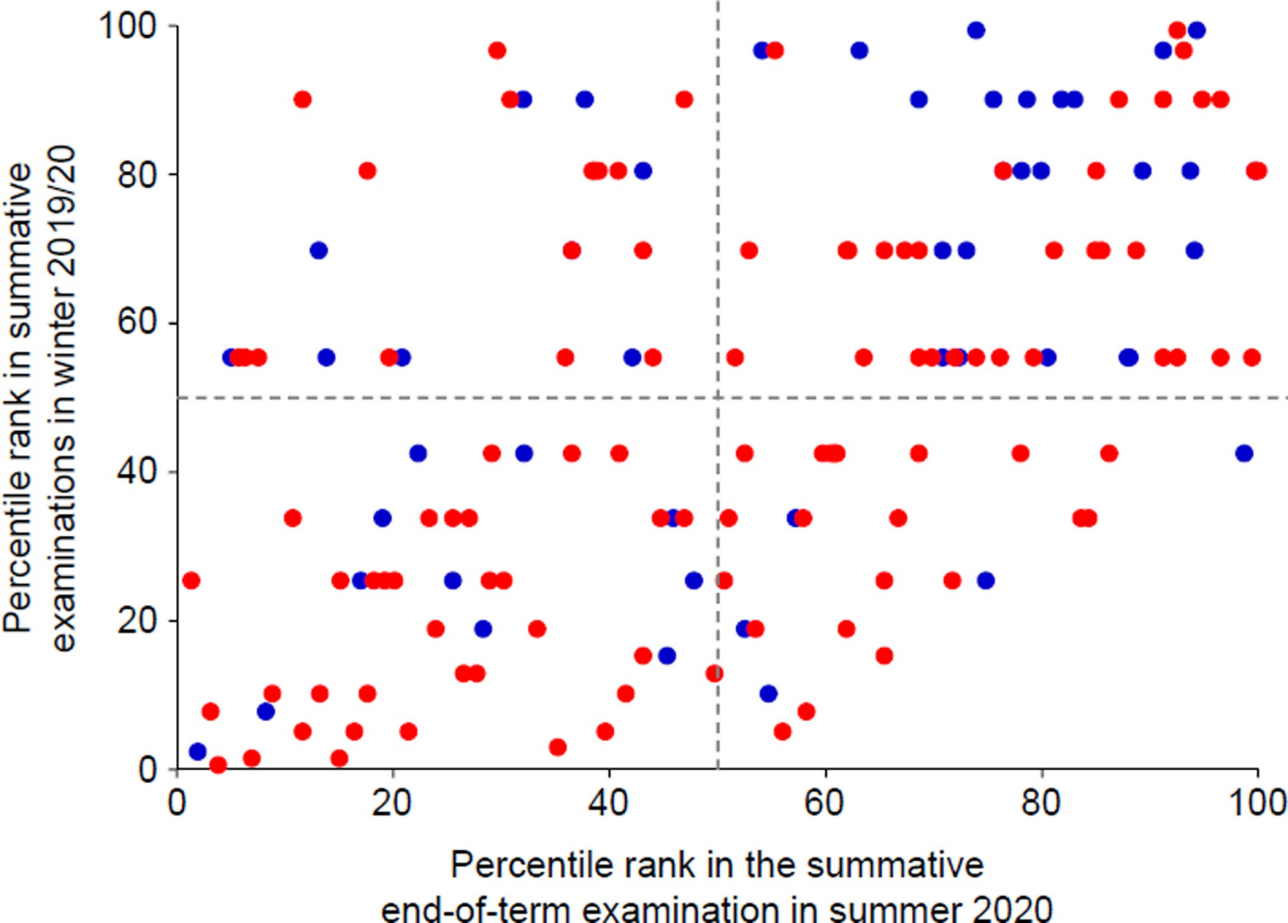
Scatterplot with percentile ranks in summative exams of the previous term vs. the summative end-of-term module in summer 2020. Red circles, female students; blue circles, male students. Splitting performance in both terms at the 50^th^ percentile yielded four distinct student groups: two groups in which students stayed in either the lower (group I: n = 46; 29.7%) or the upper half (group II: n = 57; 36.8%) of their cohort, one group in which performance had deteriorated (group III: n = 26; 16.8%) and one with an improvement of performance (group IV: n = 26; 16.8%). The proportion of female students (red circles) was non-significantly higher in the deterioration group compared to the improvement group (80.8% vs. 65.4%; p = 0.221).

Scores achieved in summative MC examinations at the end of summer term 2020 as well as the end of the preceding term were compared in terms of percentile ranks in order to identify performance trajectories in 155 students with complete exam data for both terms. The correlation between percentile ranks before and after the beginning of the pandemic explained 25% of the variance (r = 0.504; p < 0.001). While there had been a non-significant trend for female students occupying lower percentile ranks than male students in winter term 2019/20 (50.3 ± 27.8 vs. 57.3 ± 28.5; p = 0.148), this finding was more pronounced and significant in summer 2020 (46.0 ± 28.3 vs. 60.7 ± 28.2; p = 0.003) (Fig 2). Based on these findings, gender was included as an independent variable in subsequent analyses.

### Analysis 2: Association between resource use and learning outcome

In order to identify factors associated with favourable performance in either the summative MC exam (factual knowledge) or the formative gaming session (knowledge application), univariate linear regression analyses with percent score in either of the exams as dependent and student gender, previous percentile ranks and various indicators of resource use as independent variables were conducted. Variables with a significant association in the univariate analysis were entered into a multivariate linear regression. The data presented in Table 1 show that more favourable previous performance (indicated by higher percentile ranks in summative exams in the preceding term), male gender, a shorter delay to completion of and higher percent scores in e-seminars as well as a higher proportion of correct answers to #clue items were associated with higher percent scores in the summative MC exam. After adjusting for all variables in the model, only previous performance and male gender remained significant predictors of MC exam performance.

**Table 1:** Overall predictors of student performance.

Independent variables	Unadjusted beta (95% CI^a^)	Adjusted beta (95% CI^a^)
**Dependent variable: Percent Score in the summative MC examination**		
Previous exams: Percentile rank	0.15 (0.11 to 0.20)***	0.13 (0.08 to 0.18)***
Female gender	-4.05 (-6.81 to -1.29)**	-3.10 (-6.14 to -0.05)*
Assignments: Mean delay to completion	-0.06 (-0.63 to 0.31)	
e-seminars: number completed	-0.16 (-0.02 to 1.56)	
e-seminars: percent score achieved	0.21 (0.09 to 0.33)**	0.07 (-0.06 to 0.22)
e-seminars: mean delay to completion	-1.05 (-1.95 to -0.14)*	-0.55 (-1.46 to 0.37)
#clue: number of questions answered	0.03 (-0.05 to 0.11)	
#clue: percentage of correct answers	0.16 (0.05 to 0.27)**	-0.02 (-0.13 to 0.10)
#clue: mean delay to completion	0.08 (-0.70 to 0.86)	
**Dependent variable: Percent Score in the formative gaming session**		
Previous exams: Percentile rank	0.01 (-0.08 to 0.09)	
Female gender	1.65 (-3.20 to 6.50)	
Assignments: Mean delay to completion	-0.58 (-1.39 to 0.24)	
e-seminars: number completed	-0.60 (-1.99 to 0.81)	
e-seminars: percent score achieved	0.30 (0.07 to 0.53)*	
e-seminars: mean delay to completion	0.90 (-0.74 to 2.55)	
#clue: number of questions answered	0.01 (-0.13 to 0.15)	
#clue: percentage of correct answers	0.18 (-0.01 to 0.37)	
#clue: mean delay to completion	-0.83 (-2.23 to 0.58)	

^a^ confidence interval.

*p < 0.05.

**p < 0.01

***p < 0.001.

Percent score achieved in e-seminars was the only variable significantly associated with more favourable outcome on the formative serious gaming session in the univariate regression.

### Analysis 3: Early predictors of high performance

Results of the univariate and multivariate regression analyses aimed at identifying early predictors of suboptimal end-of-term summative exam performance are presented in Table 2. According to the unadjusted analysis, a delay in completing the first assignment or #clue items did not predict MC exam scores while there was an inverse relationship between delay to completion of the first e-seminar and exam performance. Percent scores in e-seminars and #clue achieved in the first week predicted percent scores in the final MC exam; after adjustment, this association persisted for performance in the first e-seminar.

**Table 2:** Early predictors of student performance.

Independent variables	Unadjusted beta (95% CI^a^)	Adjusted beta (95% CI^a^)
**Dependent variable: Percent Score in the summative MC examination**		
First assignment: delay	-0.04 (-0.02 to 0.19)	
First e-seminar: completion	-0.17 (-3.51 to 3.18)	
First e-seminar: percent score	0.22 (0.11 to 0.33)***	0.16 (0.01 to 0.30)*
First e-seminar: delay	-0.91 (-1.54 to 0.29)**	-0.67 (-1.37 to 0.03)
First week #clue: items completed	-0.01 (-0.31 to 0.31)	
First week #clue: percent score	0.10 (0.01 to 0.18)*	0.06 (-0.03 to 0.15)
First week #clue: mean delay	0.21 (-0.39 to 0.80)	

^a^ confidence interval.

*p < 0.05.

**p < 0.01

***p < 0.001.

## Discussion

In this prospective study, we found evidence of a heterogeneous use of digital learning resources. Performance in question-based tools (e-seminars, #clue) was related to consecutive summative exam performance. However, in the adjusted model, only male gender and achievement in the previous term predicted higher scores in the end-of-module examination. The main finding of the present study is that early prediction of summative exam performance is possible by paying attention to the performance in question-based tools already in the first week. This implies the possibility to identify students at risk and offer them help early on in a setting where contact teaching is not possible. The results suggesting that women might be disadvantaged in a strictly digital curriculum (based on their weaker performance in e-seminars, #clue and the final MC examination), were unexpected and might be due to confounding by unknown factors. However, given there was previously no significant difference in performance between male and female students of the same cohort, this finding warrants further investigation.

### Medical education during the corona pandemic

The corona pandemic has forced medical schools to replace most forms of contact teaching with digital formats. Many qualitative studies have examined student perceptions of these changes, including a potential impact on their own perceived learning outcome. One survey in the pre-clinical phase of medical education in California revealed that despite a positive appraisal of increased flexibility associated with remote learning, students felt that the quality of instruction had decreased [19]. Other studies described the implementation of various instructional formats such as synchronized online classes [20], flipped classroom approaches [21], case vignettes [22], ungraded tests [23] and quizzes [24]. However, most of these reports were qualitative in nature and focussed on student satisfaction and usability aspects. We could not find any studies investigating actual use of specific resources and linking student behaviour and performance during remote learning to summative exam results. Given that the detection of students at risk of failure is more difficult in the absence of contact teaching, the identification of predictors of poor performance in consecutive exams would be particularly helpful.

### Critical appraisal of the resources used in this study

Internal consistency of the two end-of-module examinations (MC questions and virtual A&E department) was acceptable, and the lack of a significant correlation between performances in these two modalities is in line with the assumption that they test different learning outcomes. Scores achieved in the end-of-module MC exam were predicted by MC exam performance in the preceding term, corroborating earlier findings of good consistency across consecutive exams addressing similar competencies [25].

Despite our attempts to align digital teaching resources and examinations to educational targets, we did not find a correlation between student performance in e-seminars and the virtual A&E department. Both were designed to address the application of knowledge to real patient cases, but the association between key feature question scores and performance in the consecutive summative MC exam in the unadjusted analysis suggests that higher-performing students are at an advantage for both question types. In agreement with our expectations, scores achieved in #clue were positively correlated with end-of-module exam scores but not with serious game performance. However, the lack of strong associations between resource use, performance in e-seminars and #clue and final exam scores in the adjusted analysis suggests that the resources used in this study did not allow a sharp distinction between knowledge acquisition and knowledge application.

### Gender differences

Our results indicate that, with regard to knowledge acquisition, female students did not benefit from using the digital resources to the same extent as did male students. In fact, female students answered more #clue questions than males but achieved lower scores. Similarly, male students outperformed their female peers in e-seminars. The significant gender difference in summative exam scores is remarkable as it was not present in the preceding term, suggesting that the shift from contact to remote learning had greater impact on female students. Especially this result was striking due to the previous studies describing male gender being a risk factor for failure in medical education [17]. Furthermore there were no significant differences in use of learning resources between male and female students in terms of frequency or delay of processing in any of the learning resources that could explain the disadvantage of women regarding knowledge acquisition. The only exception were the #clue questions, where females even answered more items than males (but fewer items correctly). One possible explanation of our findings could be that women have been reported to suffer from increased psychosocial distress related to the pandemic [26]. From the perspective of educational psychology, female students might benefit more from social interaction and joint discussions during contact teaching, the lack of which might have disadvantaged them in the digital-only curriculum. However, previous research does not indicate that digital instructional formats as such put female students at a disadvantage. In any case, the loss of a majority of significant associations in the adjusted analyses suggests that most of the univariate associations regarding knowledge acquisition were driven by differences in gender and previous performance.

### Prediction of student performance

We hypothesised that delayed completion of assignments, e-seminars and #clue questions might be predictive of suboptimal performance in the subsequent summative exam. However, neither the timing of assignment submissions and #clue question completion nor the number of completed e-seminars or #clue questions was correlated to exam scores. Univariate regression analyses suggested that faster completion of e-seminars was associated with higher exam scores, but this effect disappeared in the adjusted analysis, probably due to an overriding effect of gender and previous exam performance that would explain student behaviour with regard to e-seminars.

Our finding of some predictive value of early e-seminar scores for achievements at the end of the module is in line with earlier reports of a positive relationship between performance in teaching interventions during the semester and performance on final exams [8]. Since the other predictors of success (i.e., previous exam performance and male gender) cannot be modified, it is important to identify warning signs of potential failure. Thus, students underperforming in the first e-seminar might receive specific counselling about this association and could be offered specific help if needed.

### Strengths and limitations

To our knowledge, this is the first study prospectively assessing objective student performance in relation to the use of digital resources replacing contact teaching in medical education during the coronavirus pandemic. Strengths include the large sample size with high response rate, the implementation of various digital teaching formats aligned to different educational targets, and the granularity of the data. Owing to the nature of this observational study, there was no control group, and despite the availability of comprehensive data on learning behaviour, we cannot comment on how much time students spent on self-study or self-directed small-group discussions. Furthermore, the effects of individual components of the digital curriculum on summative exam performance cannot be disentangled.

### Future research

The differences in summative exam performance testing factual knowledge between male and female students were unique to the digital-only curriculum, and more qualitative research might help elucidate the underlying reasons. If this finding is replicated in other institutions or educational contexts, measures must be taken to counterbalance this disadvantage.

With regard to early predictors of summative exam performance, strategies to automatically identify students at risk and systems proactively offering help should be devised and validated.

## Conclusion

The association between performance in the first week and summative exams might help to identify students at risk and offering help early on. The unexpected finding of female students performing significantly worse than male students requires further study to determine whether the shift to a digital-only curriculum actually puts women at a disadvantage with regard to knowledge acquisition.

## Supporting information

S1 Dataset (SAV)
